# Iron, ESA, and HIF-Inhibitors: Are There Other Opportunities to Improve Anemia of CKD?

**DOI:** 10.3390/jcm15187070

**Published:** 2026-09-11

**Authors:** Mario Bonomini, Lucia Del Vecchio, Roberto Minutolo, Vittorio Sirolli, Francesco Locatelli

**Affiliations:** 1 Department of Medicine and Science of Aging, Nephrology and Dialysis Clinic, SS. Annunziata Hospital of Chieti, “G. d’Annunzio” University of Chieti-Pescara, 66100 Chieti, Italy; mario.bonomini@unich.it (M.B.); vsirolli@unich.it (V.S.); 2 Department of Nephrology and Dialysis, ASST Lariana, 22100 Como, Italy; 3 Nephrology Unit, Department of Advanced Medical and Surgical Sciences, University of Campania “Luigi Vanvitelli”, 80138 Naples, Italy; roberto.minutolo@unicampania.it; 4 Department of Nephrology and Dialysis, ASST Lecco, 23900 Lecco, Italy; francesco.locatelli2210@outlook.it

**Keywords:** anemia, chronic kidney disease, ESAs, iron, HIF-PHIs, monoclonal antibodies, SGLT2-inhibitor, dietary supplement, hepcidin, inflammation

## Abstract

Anemia is a common complication of chronic kidney disease (CKD) and is associated with CKD progression and other adverse outcomes, which can result in increased rates of cardiovascular events and mortality. The search for the most effective and at the same time safe therapy for anemia in patients with CKD has always been a focus for nephrologists. The traditional treatment of CKD-dependent anemia is a combination of iron supplementation (oral and intravenous) and erythropoiesis-stimulating agents (ESA). Clinical practice data on the management of anemia in CKD patients highlight that anemia treatment is still suboptimal, and treatment is frequently omitted or underused due to a high rate of therapeutic inertia in initiating and increasing doses of anemia drugs. Moreover, ESA therapy has been associated with a risk of cardiovascular events, particularly in hypo responder patients requiring high doses or at high Hb targets. More recently, a class of oral drugs (hypoxia-inducible factor prolyl hydroxylase inhibitors) has been developed based on the modulation of the mechanisms underlying HIF oxygen sensing, leading to a simultaneous stimulation of endogenous erythropoietin production and improved iron metabolism. Unexpectedly, they went from a theoretical physiological superiority to a substantial non-inferiority, and remain largely underused likely because of the questionable concerns of their long-term safety. The landscape of the management of CKD anemia is evolving, including potential therapeutic erythropoietic strategies that are available in clinical practice and new approaches under clinical development, which can be employed as standalone approaches or eventually combined with conventional anemia treatments. We review here the most recent developments in investigational strategies for increasing erythropoiesis and improving the management of CKD anemia.

## 1. Introduction

Anemia is a common complication of chronic kidney disease (CKD); its prevalence and incidence progressively increase as kidney function declines, with up to 60% of people with non-dialysis-dependent CKD and nearly 80% of dialysis patients having anemia [1,2,3,4,5]. The causes of anemia of CKD are multifactorial and include the reduced production of endogenous erythropoietin, absolute and/or functional iron deficiency, and inflammation and sub-clinical blood loss, among others [5]. Anemia is associated with CKD progression and other adverse outcomes, including worsening angina, impaired cardiac contractile function and left ventricular hypertrophy, which can result in increased rates of hospitalizations for cardiovascular events and mortality [6,7,8]. Besides a poor clinical outcome, the worsening of anemia can lead to a negative impact on the quality of life of people with CKD and their caregivers [9,10].

The traditional treatment of CKD-dependent anemia is a combination of iron supplementation and erythropoiesis-stimulating agents ESA [11]; however, in large randomized controlled trials (RCTs), ESA therapy has been associated with a risk of cardiovascular events [6,7,8], particularly in hypo responder patients requiring high doses of ESA administered to reach high hemoglobin (Hb) targets [12,13]. Despite the long-standing experience with this standard therapy, clinical practice data on the management of anemia in patients with non-dialysis-dependent CKD under nephrologist care highlight that anemia monitoring is suboptimal, and treatment is frequently omitted due to a high rate of therapeutic inertia in initiating anemia drugs [1,2,14]. More recently, a class of oral drugs (hypoxia-inducible factor prolyl hydroxylase inhibitors (HIF-PHIs)) has been developed and is in clinical use based on the modulation of the mechanisms underlying HIF oxygen sensing, leading to a simultaneous stimulation of endogenous erythropoietin production and improved iron metabolism [15]. Many large-scale RCTs have shown that HIF-PHIs, including roxadustat, daprodustat, and vadadustat, are non-inferior to ESAs in raising hemoglobin (Hb) levels [16,17,18,19]. Evidence regarding the efficacy and safety of HIF-PHIs has also emerged from several meta-analyses [20,21,22] and from real-world data from China and Japan [23,24], none of them showing relevant safety issues. However, two randomized clinical trials of patients with NDD CKD raised some safety issues [18,19]. For this reason, roxadustat was not approved for clinical use by the FDA, while it was approved by the EMA, and daprodustat and vadadustat have not been approved in NDD patients in Europe and the United States. The most recent KDIGO anemia guidelines have positioned HIF-PHIs as second-line agents relative to ESAs, primarily owing to insufficient long-term follow-up data to adequately characterize their thromboembolic, cardiovascular, and neoplastic risk profiles [25]. Despite cautions, especially in selected patient categories, the European Renal Best Practice commentary posited HIF-PHIs as possible alternatives to ESAs [26]. It is a matter of fact that these drugs remain largely underused, likely because of the questionable concerns regarding their long-term safety [25,27].

Regardless of the current positioning of HIF-PHIs relative to ESAs, the need for novel anemia management approaches in CKD patients remains unmet.

The landscape of the management of CKD anemia is rapidly evolving by including potential therapeutic erythropoietic strategies that are available in clinical practice and new approaches under clinical development. Therapies in clinical practice, which may represent complementary erythropoietic approaches to first-line treatment in CKD, are represented by pharmacologic agents and nutritional supplements. Erythropoietin (EPO) mimetic agents, activin traps, hepcidin therapeutics (hepcidin antagonists, monoclonal antibodies), as well as drugs inhibiting cytokine-dependent anemia (ziltivekimab, clazakizumab, and canakinumab) are erythropoietic strategies under clinical development, used as standalone treatments or eventually combined with conventional anemia treatments. The latter therapeutic strategy links with the well-known pathogenetic role of inflammation in inducing anemia by either blocking iron availability (higher hepcidin levels) or by blunting the differentiation and proliferation of erythroid precursors in the bone marrow [28]. It is also important to highlight that drugs now considered as cornerstones for retarding the progression of CKD and preventing cardiovascular events (sodium glucose co-transporter 2 inhibitors, SGLT2i) have a potential impact on the delay of anemia, occurrence as well as on anemia correction.

In this work, we report the most recent developments in these new potential therapeutic erythropoietic strategies for the management of CKD anemia.

## 2. Materials and Methods

A comprehensive literature search of PubMed, Web of Science, Cochrane and Scopus databases was conducted to identify studies on investigational strategies for increasing erythropoiesis and improving the management of CKD anemia, other than iron, ESAs, and HIF-PHIs. The search primarily covered publications from 2010 up to 15 March 2026, with key words including “chronic kidney disease”, “anemia”, and “erythropoiesis”. A manual search of the reference lists of included studies was also conducted, to ensure the inclusion of all pertinent investigations. Both clinical studies in humans and relevant pre-clinical studies were considered. We did not elaborate the inclusion and exclusion criteria. Study selection was based on scientific relevance and contribution to the current understanding of the field rather than on the predefined methodology required for systematic reviews. We made use of a narrative synthesis approach, considering the heterogeneity of study designs and endpoints.

## 3. New Strategies for Increasing Erythropoiesis in CKD

### 3.1. Pharmacologic Agents Available in Clinical Practice

#### 3.1.1. SGLT2 Inhibitors

Consistent evidence suggests that, among ancillary effects, SGLT2i can increase Hb levels in patients with or without CKD—an effect that cannot be attributed solely to hemoconcentration. Indeed, early plasma volume contraction may account for a transient Hb rise detectable within the first week of therapy, but could not explain the sustained increase observed over months, combined with documented rises in reticulocyte counts and a trend of increased serum EPO levels [29,30]. Further reinforcing the strength of the observation, at the end of SGLT2i therapy, Hb returns to baseline values. Furthermore, SGLT2i possibly influences iron metabolism.

Several hypotheses have been proffered to explain why SGLT2i increases Hb levels; most of them focus on erythropoietic responses driven by oxygen concentration in the kidney medulla. According to their primary mechanism of action, SGLT2i reduces sodium–glucose reabsorption in the early proximal tubule. This reduces oxygen consumption, thereby improving superficial cortical oxygenation. However, a greater fraction of sodium transport occurs distally to the S3 segment and medullary thick ascending limb with increased oxygen consumption at that level. Experimental work and modelling indicate that this redistribution increases oxygen demand and lowers oxygen tension in the deep cortex and outer medulla [31]—providing a localized hypoxic stimulus that upregulates renal erythropoietin synthesis and drives erythropoiesis. In this view, changes in regional tubular oxygen consumption act as the upstream signal that links SGLT2 blockade in the proximal nephron to the downstream anti-anemic effect observed in clinical trials [32]. A similar but divergent hypothesis proposes that SGLT2i improve iron mobilization and activate HIF-2α-driven erythropoietin production [33]. This is caused by a reduction in glucotoxicity and oxidative stress in the tubulo-interstitium, and a modulation of iron metabolism through hepcidin suppression and erythroferrone upregulation. Notably, SGLT2i also increases the expression of vascular endothelial growth factor [34].

A third mechanism includes the upregulation of nutrient deprivation signaling, especially sirtuin 1 (SIRT1). SGLT2s are considered as caloric sensors; their inhibition promotes a state of starvation mimicry, which is characterized by a metabolic shift from glucose to lipid and ketone utilization. This signaling reduces glucotoxicity and oxidative stress, improves mitochondrial biogenesis and function, and reduces inflammation that normally upregulates hepcidin. Moreover, SIRT1 has a direct effect on the activity of HIF-2α during hypoxia, favoring HIF-2α–mediated erythropoietin production in the kidney (and partly liver) [35].

Finally, by attenuating tubulo-interstitial injury and fibrosis, SGLT2i may favor a microenvironment in which otherwise dysfunctional renal interstitial fibroblasts could revert toward an EPO-producing phenotype, although this is at present speculative, since direct experimental evidence for this phenotype switch is lacking.

Some of the above mechanisms have strong connections with those explaining at least part of the nephron- and cardiovascular-protective effects of SGLT2i.

The erythropoietic effect of SGLT2i is observed across randomized controlled trials spanning different cardiorenal populations and in the presence or absence of anemia at baseline.

Back in 2020, a post-hoc analysis of the CREDENCE trial showed a mean Hb gain of 0.7 g/dL in patients randomized to canagliflozin compared to placebo, together with a lower rate of anemia event or initiation of treatment for anemia (hazard ratio 0.65; 95% CI 0.55–0.77; *p* < 0.0001) [36]. Canagliflozin also influenced iron metabolism, with an 11.5% decrease in serum ferritin and a 2.1% increase in TIBC compared with placebo, with no clear effect on serum iron or transferrin saturation [37]. The analysis was limited by the fact that iron parameters were measured at baseline only in two thirds of the patients.

A pooled analysis of 14 placebo-controlled trials enrolling 5325 patients with type 2 diabetes showed that dapagliflozin also produced a mean Hb increase of 0.81 g/dL in anemic patients, with anemia correction achieved in 52% versus 26% under placebo at six months [38].

Differing from CREDENCE, the DAPA-CKD trial enrolled a mixed population (67.5% with type 2 diabetes). Nearly 40% of the trial population was anemic at baseline (Hct less than 39% in men and less than 36% in women). Dapagliflozin increased Hct by 2.3 percentage points more than placebo, and corrected anemia above the Hct cut-off in 53.3% versus 29.4% [39]. These effects were consistent irrespective of baseline anemia status, diabetes, or degree of renal impairment.

In EMPA-KIDNEY, empagliflozin had a modest effect on Hb and Hct consistent with the erythropoietic effects observed with SGLT2i in other CKD trials, although detailed anemia-specific endpoints have not yet been separately reported [40].

A meta-analysis of 14,748 patients with type 2 diabetes confirmed a class effect, with a weighted mean increase of 0.56 g/dL, with canagliflozin possibly the more potent agent for the anti-anemic effect [41]. This possibly reflects its comparatively lower selectivity for SGLT2 over SGLT1 [42]—a dual inhibition that may amplify the downstream erythropoietic response relative to more selective agents.

The data from heart failure trials are in line with those observed in type 2 diabetes or CKD. In the EMPEROR-Reduced trial, empagliflozin raised Hb by 0.35 g/dL at week 4 and 0.69 g/dL at week 12, with the effect disappearing within one month of discontinuation [43]. In DAPA-HF, 22% of the patients were anemic at baseline; dapagliflozin therapy corrected anemia in 62.5% versus 41.1% on placebo [44]. This was observed even in the absence of iron supplementation in more than 90% of patients, including those meeting criteria for iron deficiency—raising the possibility that SGLT2i may partially circumvent the iron-restricted erythropoiesis typical of cardiorenal disease. In line with this observation, empagliflozin significantly reduced hepcidin levels in the Empire HF trial [45].

A recent systemic metanalysis considered the anti-anemic effects of dapagliflozin across different patient populations [46]. Compared with placebo, dapagliflozin nearly doubled the likelihood of anemia correction (RR 1.83; 95% CI 1.47–2.26) while reducing new-onset anemia incidence by 78% (RR 0.22; 95% CI 0.08–0.60). The effect was consistent across all studied populations, but more evident in the setting of heart failure, followed by CKD and diabetes.

Interestingly, recent post-hoc analyses of SGLT2i-outcome trials suggest that changes in Hb and Hct may mediate a substantial proportion—up to roughly one-third—of the overall cardiorenal benefit in certain patient populations [47,48].

Finally, a recent pre–post observational analysis in CKD patients (stages 1–4) treated with SGLT2i for 18 months confirmed a possible favorable effect of those drugs on anemia-related parameters [49].

In some patients, the Hb/Hct increase secondary to SGLT2i may exceed the upper normal limits, and sometimes discloses a masked diagnosis of polycythemia vera, especially in the presence of accompanying signs (iron deficiency, leukocytosis, thrombocytosis, pruritus, or splenomegaly). Conversely, SGLT2i therapy could mask pathological states causing anemia. That said, the Hb/Hct increase also over the normality range has not been found to be significantly associated with an increased thrombotic risk, as shown by recent real-world studies [50,51].

In conclusion, the erythropoietic effect of SGLT2i represents a consistent and clinically meaningful finding across cardiorenal populations, independent of the underlying mechanism—which remains to be fully elucidated. From a clinical standpoint, these agents significantly raise Hb levels and correct anemia, and may thereby contribute to the cardiorenal benefit observed in landmark trials. At present, their role in combination with other anti-anemic drugs (ESAs, HIF-PHI, oral and IV iron) is unknown.

#### 3.1.2. Other Adjunct Drug Therapies

*Pentoxifylline* (oxpentifylline) is a non-selective phosphodiesterase inhibitor developed as a treatment for peripheral vascular diseases due to its hemorheological properties, which also exhibits important anti-inflammatory and antioxidant properties [52].

Daily treatment with 400 mg orally of pentoxifylline in 7 CKD anemic [53] and 12 ESRD patients with EPO-resistance [54] significantly increased Hb levels, and decreased serum tumor necrosis factor-alpha (TNF-α) [53] and the ex vivo T cell generation of TNF-alpha and interferon gamma [54]. The Hemoglobin elevation in Erythropoietin Resistance with Oxpentifylline (HERO) trial assessed pentoxifylline treatment in patients with CKD stage 4 or 5 (including dialysis patients) and ESA-hyporesponsive anemia, randomized to receive either placebo or pentoxifylline (400 mg daily) orally for 4 months [55]. The primary outcome of the study was the ESA resistance index at 4 months. As compared to the control group, pentoxifylline did not significantly modify ESA hypo-responsiveness or ESA dose. Among secondary outcomes, pentoxifylline significantly increased Hb concentration relative to the control group [55]. Finally, a recent systematic review and meta-analysis including 19 studies demonstrated that the use of pentoxifylline safely increased Hb levels. Of note, pentoxifylline was associated with a significant improvement in eGFR and reduction in CRP and TNF-α. The efficacy seems related to treatment duration and dosage [56].

The improvement of Hb concentration by pentoxifylline therapy may occur via the inhibition of proinflammatory cytokine production [53,54]. A novel mechanism has been suggested by a recent randomized study in 80 anemic patients on HD [57]. In the pentoxifylline-treated group (400 mg of pentoxifylline twice daily for 6 months), an appreciable increase in Hb (from 9.7 to 10.8 g/dL) was observed after one month of administration and maintained over the study course, along with a decrease in ESA dose and in serum levels of TGF-β1 and hs-CRP. Notably, HIF-2α increased significantly at the end of the pentoxifylline intervention, which might explain the amelioration of anemia [57].

A beneficial therapeutic effect for CKD patients may be obtained by combining pentoxifylline with folic acid. In a prospective trial, CKD stage 3–5 patients were randomized into four groups (20 patient/group) and followed up for 6 months: the control group received standard usual care therapy only, while the other groups received pentoxifylline (400 mg twice daily) and/or folic acid (500 mcg once daily) added to standard usual care therapy [58]. In the three intervention groups compared to the control group, statistically significant increases were found in Hb levels and eGFR, with a decrease in urinary protein-to-creatinine ratio. Notably, a synergistic improvement in all the measured parameters was observed in the pentoxifylline + folic acid group [58].

*L-carnitine* is a naturally occurring compound that is involved in the intermediary metabolism essential for mammalian bioenergetic processes [59]. The anti-anemic action of L-carnitine is based on its biophysical, metabolic and anti-apoptotic effects on erythropoiesis and the function of circulating RBCs [60]. The L-carnitine evidence is of limited quality and heterogeneity. Despite clinical studies suggesting that L-carnitine supplementation can alleviate anemia in HD patients, some controversy about its use in this indication persists. However, a recent meta-analysis comprising 18 trials with 1090 patients on HD showed that L-carnitine administration significantly improved the response to ESA and reduced the required ESA dose while maintaining Hb and hematocrit levels [61]. Furthermore, a recent small, randomized, double-blind study in 20 HD patients with ESA hypo-responsiveness showed that supplementation with L-carnitine for 3 months induced a 25% reduction in erythropoietin resistance index, measured as an indicator of treatment effectiveness, supported by a 42% reduction after 6 months of treatment in the secondary validation conducted in an independent dataset [62].

*Androgen* deficiency, defined by low testosterone levels, has a high prevalence in CKD [63,64]. Testosterone, the cornerstone therapy for men with hypogonadism, may have various favorable effects on the hematopoietic system [65]. A systematic review and meta-analysis including 9 cohort studies identified low endogenous testosterone as an independent predictor of adverse clinical events among male patients with CKD (mainly dialysis patients) [66]. A more recent cross-sectional observational study in 322 CKD stage 4 and 5 (including dialysis) patients reported correlations of lower Hb and higher ESA dose with lower testosterone levels [64].

However, few intervention studies have evaluated the role of androgen replacement in treating CKD anemia. A Cochrane review of 2014 including eight small RCTs assessing the use for 6 months of synthetic analogues of testosterone (oxymetholone, nandrolone) in adult CKD patients showed insufficient evidence to confirm that the use of androgens is associated with substantial benefits [67]. Limited data are available for native testosterone. Two RCTs in HD patients failed to show significant improvements in anemia management by transdermal [68] or intramuscular testosterone administration [69]. However, a retrospective chart review showed increased Hb concentrations and reduced ESA doses in hypogonadal men on HD treated with testosterone [70]. Moreover, the findings of a nested trial from the TRAVERSE study group, which also included stage 3 CKD patients, demonstrate the correction of anemia with transdermal testosterone [71]. A critical need persists for larger, high-quality prospective studies to comprehensively investigate the long-term safety and efficacy of testosterone therapy for CKD anemia [72].

### 3.2. Nutrition Interventions

Several dietary approaches have been examined for their potential effect on the management of anemia in CKD (Table 1).

**Zinc.** Zinc is an essential trace element necessary for the activity of more than 300 enzymes, and it has various physiological effects, including playing a role in erythropoiesis. Blood zinc levels in patients with CKD, and on HD are significantly lower as compared to healthy controls [73]. Zinc deficiency in dialysis has been associated with a reduced responsiveness to ESA [74]. Zinc deficiency may arise from multiple factors [75], and it is increasingly recognized as a contributor to renal anemia [76].

Oral zinc supplementation appears to be a feasible treatment approach for zinc-deficient CKD anemic patients treated with ESA (Table 1). The administration for 12 months of polaprezinc, an anti-ulcer agent containing zinc (zinc L-carnosine), significantly reduced ESA dosage and erythropoietin responsiveness index [77], and improved Hb levels while significantly reducing the dosage of erythropoietin [78]. Notably, the anti-anemic use is off-label. Another zinc-containing compound, zinc acetate hydrate, significantly reduced both ESA dosage and ERI in 21 anemic HD patients [79]. Furthermore, a recent retrospective analysis in populations with zinc deficiency and chronic anemia showed that supplementation with oral zinc sulfate significantly increased Hb levels in CKD 3–5 patients [80]. In all these studies, the improvement of anemia management was associated with an increase in serum zinc levels.

Overall, zinc supplementation may be a useful adjunctive therapy in CKD anemia [80]. However, the administration of zinc preparations may require the monitoring of serum zinc and copper levels. Indeed, excessive zinc supplementation may induce acquired copper deficiency [81], as zinc levels above the physiological range competitively inhibit intestinal copper absorption [74]. Accordingly, zinc-induced copper deficiency has been reported in patients on HD, and can induce resistance to ESA treatment, pancytopenia, or myelopathy [74,79,82], though it is often overlooked as a cause of anemia [74]. Whether zinc supplementation is a safe and effective therapeutic strategy for improving anemia in patients with CKD requires to be established by further RCTs.

**Lactoferrin**. Lactoferrin is a milk derivative glycoprotein that constitutes a source of iron and has anti-inflammatory properties. Lactoferrin can decrease IL-6 secretion, leading to hepcidin downregulation, thereby improving the utilization of iron and the erythropoietic process [83].

The effects of lactoferrin on iron deficiency anemia were evaluated in a randomized trial conducted in HD patients receiving iron saturated lactoferrin or ferrous glycine sulfate orally twice a day for 6 months. Both treatments induced a decrease in serum hepcidin levels with an increase in Hb and transferrin saturation. In the lactoferrin treatment arm, there was a significantly higher magnitude of change in these parameters. The effectiveness of lactoferrin compared to simple iron supply seems most strongly related to an anti-inflammatory mechanism [84].

In a more recent study, adult patients with stage 5 CKD (85% on dialysis) on stable ESA dosage were treated for 1 month with oral lactoferrin twice daily, with or without iron supplementation [85]. A slight but significant improvement in Hb levels was observed from baseline, particularly in patients receiving lactoferrin with iron supplementation. The study suffers from several limitations, including the short duration, absence of randomization, and lack of data on adverse effects and iron metabolism parameters [85]. Thus, the role of oral lactoferrin in anemic CKD patients remains to be elucidated.

**Gut microbiota modulators**. Gut microbiota modulation may represent a potential approach to improving renal anemia, as gut dysbiosis is common in CKD and dialysis, and may contribute to inflammation, toxin accumulation, and ESA hypo-responsiveness [86,87,88]. Strategies to modulate gut microbiota include dietary interventions and the use of probiotics, prebiotics, and synbiotics, aiming to restore microbial balance and reduce systemic inflammation [88]. Probiotics are non-pathogenic microorganisms with anti-inflammatory effects, which significantly enhance the absorption of iron. Prebiotics are functional food ingredients that facilitate beneficial gut bacteria and may improve iron absorption and anemia. Synbiotics are a combination of probiotics and prebiotics.

Some studies indicate that biotics potentially play roles in improving anemia in ESRD patients (Table 1). A prospective study examining the safety and efficacy of synbiotics supplementation in HD patients reported, among secondary parameters, an increase in Hb and hematocrit, which proved significant in responders [89]. A randomized study in HD patients assigned to receive probiotics, synbiotics, or placebo for 12 weeks showed a significant, increasing trend of Hb in biotic-supplemented groups [90]. Moreover, the prebiotic effects of soluble dietary fiber (DF) mixture could ameliorate anemia in maintenance HD patients as compared to placebo [91]. By modulating the prebiotic activity of gut microbiota and short-chain fatty acids, DF might improve renal anemia through iron metabolism and/or EPO-related pathways [91]. Finally, a randomized placebo-controlled trial in patients on HD found that synbiotics supplementation, compared to placebo, significantly improved Hb, hematocrit, RBC count, and transferrin saturation, with a significant decrease in total iron-binding capacity [92].

A recent systematic review and meta-analysis including seven studies (three focusing on iron deficiency anemia, four examining CKD including HD), comprising eight RCTs with 632 participants, demonstrated that probiotics, prebiotics, and synbiotics may significantly increase the levels of Hb (primary outcome), improving anemia management [93]. However, heterogeneity likely stemming from variations in intervention types was high, resulting in moderate-quality evidence of Hb improvement [93]. The efficacy and safety of biotic supplementation in managing anemia in CKD remains to be elucidated by high-quality, large-scale trials.

**Natural source polysaccharides**. Natural source polysaccharides are a class of bioactive molecules that have shown therapeutic potential in anemia management [94].

Angelica sinensis has hematopoietic, anti-inflammatory, and immunomodulatory properties, which are mainly related to one of its main active ingredients, angelica sinensis polysaccharide [95]. Evidence related to CKD is, however, limited to an experimental study [96] and to a single case report [97]. The effects on renal anemia of Danggui Buxue Decoction, a traditional Chinese medicine consisting mainly of Radix angelica sinensis and Radix astragali, were evaluated in a systematic review and meta-analysis including seven randomized controlled trials and 460 dialysis patients [98]. The overall methodological quality of the included studies was poor, hence the beneficial effects and safety of the proposed therapeutic approach remain to be established [98]. Finally, in an experimental CKD rat model, the oral administration of Jujube polysaccharides, one of the active ingredients in dietary fruit of Ziziphus jujuba, improved anemia parameters and ameliorated the renal pathological injury [99].

**Other investigated adjuncts**. Vitamin C (ascorbic acid) is an antioxidant that plays a key biochemical role in iron metabolism [100]. A recent systematic review and meta-analysis assessed the impact of vitamin C supplementation as an adjunct to EPO therapy in adult hemodialysis patients with anemia [101]. Following PRISMA guidelines, 14 studies with a cumulative sample size of 650 patients could be included. Vitamin C supplementation was associated with a modest but significant increase in Hb (mean difference 0.94 g/dL) and transferrin saturation; the ferritin levels showed a slight but significant reduction along with a decrease in total iron-binding capacity. EPO requirements expressed as units/kg/week were significantly reduced. These findings are biologically possible, and may be explained by enhanced iron mobilization and utilization induced by vitamin C. However, several limitations have been underscored, and current evidence remains insufficient to support the routine use of vitamin C in the management of anemia in HD patients. Adequately powered RCTs with standardized dosing regimens, long follow-ups, rigorous safety assessments and patient-centered outcomes are needed to determine whether biochemical improvements related to vitamin C translate into meaningful clinical benefits [101].

**Table 1:** Studies with nutrition interventions in CKD anemia.

Agent	Mechanism of Anti-anemic Action	Study Design (Ref. )	Participants	Intervention	Study Duration	Main Results	Safety
Polaprezinc	Regulation of RBC production and maturation, anti-oxidative protection, involvement in heme biosynthesis	Prospective, open-label, randomized, parallel-group trial [77]	Patients on maintenance HD (*n* = 70; 35 for each study group) with low serum zinc levels (<65 mcg/dL)	Oral daily polaprezinc (150 mg; 34 mg elemental zinc) or placebo	12 months	No change within or between groups in Hb levels; no change in serum iron and transferrin saturation, decreased serum ferritin; significant decrease in ESA dosage and ERI in zinc-treated group	No reporting on adverse effects
Polaprezinc	As above	Prospective, open-label trial [78]	Patients on maintenance HD with low serum zinc levels (<80 mcg/dL)	Oral twice daily polaprezinc (150 mg; 34 mg elemental zinc) (*n* = 58) or placebo (*n* = 38)	12 months	In zinc-treated group, improvement of Hb levels, and significant decrease in ESA dosage	Two patients had diarrhea, improving following discontinuation
Zinc acetate hydrate	As above	Prospective, open-label, interventional study [79]	Patients on maintenance HD with low serum zinc levels (<60 mcg/dL)	Oral daily 50 mg zinc acetate hydrate (*n* = 21)	12 months	No change in Hb levels; significant decrease in ESA dosage and ERI	No reporting on adverse effects
Zinc sulfate	As above	Retrospective study [80]	Patients with CKD stage 3–5 (*n* = 53) with low serum zinc levels (<65 mcg/dL)	Oral daily zinc sulfate 220 mg	12 months	Significant improvement of Hb levels	No reporting on adverse effects
Lactoferrin	Decreased IL-6 secretion with hepcidin downregulation, improving iron utilization	Randomized controlled trial [84]	Patients on maintenance HD (*n* = 140; 70 for each study group) with iron deficiency anemia	Oral twice daily iron saturated lactoferrin (100 mg) or ferrous glycine sulfate (576 mg)	6 months	Both treatments increased Hb levels and transferrin saturation, and decreased serum hepcidin; significantly higher magnitude of changes in lactoferrin group	No reporting on adverse effects
Lactoferrin	As above	Prospective, observational, single-arm pilot study [85]	Stage 5 CKD patients (85% on dialysis) on stable ESA dosage, with or without iron supplementation (*n* = 46)	Oral lactoferrin (100 mg) twice daily	4 weeks	Significant improvement in Hb (g/dL) from baseline (8.18) at week 2 (8.54) and week 4 (8.96), improvement being greater in patients receiving lactoferrin with iron supplementation	No reporting on adverse effects
Synbiothics	Anti-inflammatory effect	Prospective, open-label, interventional study [89]	Patients on twice weekly maintenance HD (*n* = 38)	Oral synbiothic supplements (Nitrophage Forte ™) twice daily	6 weeks	Decrease in serum hs-CRP and TNF-alfa and increase of Hb and Hct, significant in responders	No significant adverse effects reported
Synbiotics	Anti-inflammatory effect	Double-blind, placebo-controlled, randomized trial [92]	Patients on maintenance HD (*n* = 23 in each group)	Two capsules of synbiotic supplement or placebo once a day	8 weeks	Significant increase in biotic groups of Hb, Hct and transferrin saturation, significant decrease in TIBC	Good safety reported
Synbiothics and Probiotics	Anti-inflammatory effect	Double-blind, placebo-controlled, randomized trial [90]	Patients on maintenance HD (25 for each study group)	Synbiotics (15 g prebiotics, 5 g probiotics), probiotics (5 g) or placebo 4 times daily	12 weeks	Significant increasing trend in Hb levels and improvement in mental health in biotic-treated patients	No adverse effects noted
Dietary Fibers	Modulation of the prebiotic activity of gut microbiota and short-chain fatty acids improving iron metabolism	Placebo-controlled, randomized [91]	Patients on maintenance HD (*n* = 162; 81 for each study group)	Oral daily 10 g dietary fibers mixture or placebo (potato starch)	8 weeks	Significant increase in Hb levels, the primary outcome of the study, in dietary fibers-treated patients (on average > 20%), together with increases in serum iron and ferritin concentration	No reporting on adverse effects
Angelica Sinensis	Restoration of EPO receptor signaling; enhanced production of EPO; suppressed hepcidin expression	Pre-clinical study in vivo [96]	Rat model of adenine-induced CKD	Angelica sinensis polysaccharide (0.5 or 1 g/kg/day) by oral gavage	8 weeks	Intervention normalized Hb levels, improved serum EPO levels, increased iron availability, and attenuated renal histopathological damage	No reporting on adverse effects
Angelica Sinensis	As above	Case report [97]	A HD patient with ESA resistance self-initiating consumption of herbal tea	Once weekly herbal tea containing about 12 g Radix angelica sinensis	5 months	Improvement of anemia, marked decrease in the amount of ESA administered	Episodes of intradialytic hypotension
Jujube polysaccharides	Regulation of EPO production and short-chain fatty acids release	Pre-clinical study in vivo [99]	5/6 nephrectomy CKD rat model	Jujube polysaccharides 1.2 g/kg/day by oral gavage	90 days	Administration of Jujube polysaccharides improved anemia parameters (Hb, Hct, RBC count) and ameliorated the renal pathological injury	No reporting on adverse effects

CKD, chronic kidney disease; RBC, red blood count; HD, hemodialysis; Hb, hemoglobin; ESA, erythropoiesis stimulating agent; ERI, erythropoietin resistance index; IL-6, Interleukin-6; hs-CRP, high sensitivity C-reactive protein; TNF, tumour necrosis factor; Hct, hematocrit; EPO, erythropoietin; g, gram; kg, kilogram; TIBC, total iron-binding capacity.

A recent systematic review and meta-analysis examined the clinical efficacy of vitamin D supplements for CKD patients with vitamin D deficiency and anemia [102]. Vitamin D has multiple biological effects, including transcriptional repression of the gene encoding hepcidin, anti-inflammatory and immune-modulatory effects, direct stimulation of erythropoiesis in the bone marrow, and potential support for endogenous erythropoietin production [103]. The meta-analysis included 12 RCTs comprising 779 patients—383 patients in the experimental group (with vitamin D supplementation) and 396 patients in the control group (without vitamin D supplementation). Meta-analysis showed that vitamin D supplements effectively mitigated vitamin D deficiency and could significantly increase Hb levels and reduce the dosage of erythropoietin. However, these findings remain to be validated by largescale, multicenter, and prospective RCTs [102].

Vitamin E is a fat-soluble nutrient recognized for its antioxidant [104] and “nonantioxidant” [105] properties. Vitamin E can protect the erythrocyte membrane against peroxidation, thereby increasing red blood cell survival. However, a recent paper by an expert group showed moderate evidence of a causal association between vitamin E and the development of anemia [106]. There is insufficient evidence to make recommendations on vitamin E intake in non-dialysis CKD patients, also considering that the prevalence of vitamin E deficiency is unclear, and there is some concern regarding the high-dose supplementation of vitamin E given the potentially increased risk of hemorrhagic stroke and impaired platelet aggregation [107]. In HD patients, the supplementation of vitamin E (400 IU/day) for 2 months did not increase Hb, hematocrit or RBC [108]. A better application of vitamin E in patients on HD may be represented by the use of vitamin E-modified hemodialyzers, which may improve ESA resistance and hence the anemia status, an issue that deserves further study [109].

With regard to folic acid supplementation, while it remains relevant in patients with documented or suspected folate deficiency or selected cases of ESA hyporesponsiveness, the potential benefit of folic acid supplementation in CKD patients without overt folate deficiency remains uncertain. The available evidence is limited to older, small studies in HD patients receiving erythropoietin. Although one study suggested that folic acid supplementation may improve erythropoietic responsiveness to recombinant human EPO despite normal serum folate concentrations [110], other studies found no additional hematological benefit in patients with adequate folate status [111,112]. Notably, high-dose folic acid improved EPO responsiveness in folate-deficient patients, but not in patients with normal red-cell folate levels [112]. Folic acid is potentially useful when deficiency or increased functional requirement is present. However, current evidence does not support routine folic acid supplementation as an erythropoietic strategy in folate-replete CKD patients.

### 3.3. Erythropoietic Strategies in Clinical Development

Erythropoietic strategies under clinical development include EPO mimetic peptides, activin traps (Table 2), and hepcidin therapeutics.

#### 3.3.1. EPO Mimetic Peptides

EPO mimetic peptides (EMPs) are a group of chemical compounds that, despite lacking sequence homology with EPO, can stimulate erythropoiesis via activation of the EPO receptor. *Peginesatide* (originally named Hematide; Affymax/Takeda) was the sole EMP approved by the Food and Drug Administration (FDA) in 2012 for treating adult anemic patients receiving dialysis. Though it proved effective in correcting anemia [117], unexpected fatal anaphylaxis and hypotension led to its withdrawal from the market [118].

*Pegmolesatide* (originally named pegol-sihematide, EPO-018B; Hansoh Pharmaceutical Group, Shanghai, China) is a novel synthetic pegylated EMP incorporating in its manufacturing process several improvements, and it has demonstrated a similar activity to peginesatide in vitro and in vivo, with reduced immunogenicity and a longer half-life [119].

Phase II studies in anemic CKD patients not dialyzed and on dialysis (*n* = 62 for each study group) showed significant dose-dependent increases in Hb levels and a prolonged response to subcutaneous administration. Treatment was well tolerated and adverse effects proved mild [113]. A randomized, phase III clinical trial in 347 anemic patients on dialysis demonstrated the non-inferiority of pegmolesatide as compared to epoetin alfa in increasing and maintaining Hb levels during the 52-week study. Pegmolesatide was well tolerated, and its safety profile was comparable to that of epoetin alpha [114]. Similar results in terms of safety and efficacy were observed in a randomized, noninferiority phase III study conducted across 38 centers in China in 173 NDD CKD patients with anemia [115].

These results indicate a potential effective and safe treatment for CKD anemia, allowing for less frequent dosing for the prolonged erythropoietic activity of pegmolesatide. However, they need to be confirmed in longer-term studies, and also conducted in ethnic groups other than the Asian population.

#### 3.3.2. Activin Traps

Activins are members of the transforming growth factor-beta (TGF-β) superfamily that may influence late erythropoiesis by affecting erythroid progenitor or precursor cells, or by altering bone marrow accessory cells [120]. *Sotatercept* (ACE-011; Acceleron and Celgene Corporation) is an activin receptor type IIa-IgG1 fusion protein trap that binds to circulating activin and other members of the TGF-β superfamily [121], which exerts several biologic effects, including erythropoietic activity [122]. The drug is currently under investigation in clinical and mechanistic studies for its potential use in the treatment of various hematological disorders, including anemia [123].

Two phase II, randomized studies evaluated the safety and pharmacologic effects of sotatercept on Hb levels in anemic HD patients with ESA hypo-responsiveness [116]. In the REN-001 study, after ESA washout, the target Hb response (>10 g/dL) was more frequently achieved in patients treated with sotatercept than placebo. The results regarding the Hb levels in the REN-002 trial evaluating the effects of sotatercept after switching from a prior ESA proved quite similar to those for REN-001, and were generally better in patients treated subcutaneously compared to intravenously. In both studies, sotatercept showed clinically acceptable safety and tolerability profiles, the most common treatment-emergent adverse effect being hypertension.

REN-001 also assessed the effects of sotatercept on bone mineral density and vascular calcification [116]. Namely, activin receptors were stimulated in the skeleton, vasculature, and heart during CKD [124], and the use of sotatercept might ameliorate CV- and bone disease-related CKD. Sotatercept showed a dose-dependent trend in slowing the progression of abdominal aorta vascular calcification, while the effects on bone mineral density endpoints were less consistent [116].

Current evidence regarding the use of sotatercept in CKD is derived from a small number of patients treated for a relatively short time. In addition, being nondialyzable and having a low tissue distribution and long half-life, sotatercept requires dose adjustment during use to avoid excessive accumulation leading to adverse reactions [123]. However, no further clinical development has been planned for the CKD population.

Luspartecept is another molecule of the class, but its use in CKD has not been investigated, as nearly 10% of the patients receiving the drug may develop kidney damage [125]. A novel modified activin receptor type IIa soluble ligand trap, *Elritercept* [KER-050; Berkshire Sterile Manufacturing, Lee, MA, USA], improved anemia in a recent study conducted in healthy postmenopausal women [126], but has not yet been investigated in CKD. Considering that elritercept also increases platelet levels, it could have the potential effect of increasing thrombotic risk in the setting of CKD.

#### 3.3.3. Hepcidin Therapeutics

Given the role of elevated hepcidin levels in the pathophysiology of anemia as a chronic disease, hepcidin-lowering strategies may be a clinically relevant option for treating this entity in CKD (Table 3).

Two phase I randomized trials examined the safety and tolerability of a single intravenous infusion in anemic HD patients of the hepcidin antagonist PRS-080#22 (Pieris Pharmaceuticals, Boston, MA, USA) [127], a pegylated anticalin protein that specifically and efficiently binds hepcidin [128]. Treatment was well tolerated and safe. Increased serum iron concentration and transferrin saturation and decreased free hepcidin levels were found. A randomized, phase IIa trial with PRS-080#22 enrolling 11 anemic patients on HD has been completed (NCT03325621), but the results were not made public. No further clinical development has been planned since then.

Other therapies involve the use of monoclonal antibodies targeting the hepcidin-ferroportin pathway [129]. The administration of a single dose of LY3113593 to HD patients receiving usual doses of ESA and iron induced an increase in Hb concentration together with a reduction in ferritin and hepcidin levels [129]. Clinical development was however, stopped. In the LY2928057 study, the prespecified level of Hb maintenance was not met, with a decrease over time in Hb levels, in HD patients discontinuing ESA and/or iron therapies at time of randomization. Accordingly, the development of the molecule was halted.

An additional therapeutic option may be represented by the augmentation of erythroferrone, a hormone produced by red cell precursors that inhibits the BMP signaling required for the transcription of hepcidin [130], linking erythropoiesis and iron homeostasis. In a CKD mouse model, the transgenic overexpression of erythroferrone was associated with favorable antianemic effects. This proof-of-concept study provides insights into the therapeutic potential of using erythroferrone in CKD anemia [131].

A further novel link between iron homeostasis and erythropoiesis has recently emerged from pre-clinical studies on fibroblast growth factor 23 (FGF23) [132]. Besides its classical production by osteoblasts/osteocytes, FGF23 is also produced and secreted by erythroid cells in response to iron deficiency, anemia and EPO stimulation [133,134]. Experimental evidence indicates that locally produced intact FGF23 may act as a paracrine negative regulator of erythropoiesis by activating FGF receptor 1 in erythroid progenitors, thereby impairing their differentiation and promoting mitochondrial dysfunction and oxidative stress [132]. Notably, the erythroid-specific deletion of Fgf23 in a mouse model of progressive CKD partially prevented the development of anemia and improved iron availability. These findings suggest a previously unrecognized feedback mechanism whereby erythroid-derived FGF23 may restrain erythropoiesis and limit further iron utilization under iron-restricted conditions. While potentially adaptive during acute iron deficiency, the persistent activation of this pathway in CKD may contribute to ineffective erythropoiesis. The selective modulation of erythroid FGF23 production or local FGF23 receptor1 signaling may therefore represent a novel therapeutic concept for improving erythropoietic efficiency and iron utilization, although this possibility remains entirely pre-clinical. A further investigation strategy targets transferrin receptor 2 (Tfr2), an activator of hepcidin production in the liver and a modulator of EPO signaling in erythroid cells [135]. In a mouse model of CKD, the simultaneous deletion of Tfr2 in the liver and in the hematopoietic compartment induced sustained anemia amelioration [136]. At present, however, an approach to the pharmacological inhibition of Tfr2 is lacking.

More recently, a fully human anti-BMP6 antibody (KY1070) proved able in a CKD mouse model to reverse hepcidin-mediated iron restriction and ameliorate iron availability and anemia [137]. The combination of KY1070 and darbepoetin alfa, as compared to monotherapy, was associated with a markedly better erythroid response and required a lower ESA dose [137].

Targeting hemojuvelin (a co-receptor of the BMP signaling pathway) has potential utility as a novel tool for the treatment of anemia and inflammation by lowering hepcidin levels [138]. The human monoclonal antibody DISC-0974 binds to hemojuvelin and reduces the expression of the hepcidin gene [139]. Preliminary findings have shown meaningful, dose-dependent, and sustained reductions in hepcidin in patients with myelofibrosis and anemia [140]. A randomized trial in anemic NDD CKD patients treated with subcutaneous doses of DISC-0974 had been completed (NCT05745883). The initial data show sustained reductions in serum hepcidin with a corresponding doubling of TSAT [141], but no further clinical development has been planned for the CKD population.

At present, the only molecule targeting hepcidin with the potential for further development in CKD is sevuparin. It is a low-molecular-weight heparinoid with markedly attenuated anticoagulant activity and with immunomodulatory, anti-adhesive, anti-aggregate effects. In addition to that, it has a strong hepcidin suppression effect enacted via interference with BMP/SMAD and IL-6 signaling [142]. A phase 2 study is underway in Italy that is enrolling CKD patients.

**Table 3:** Studies on hepcidin therapeutics in CKD.

Agent (Ref.)	Mechanism of Action	Study Design	Participants (Number)	Intervention, Study Duration	Main Results	Safety
PRS-080#22 pegylated anticalin protein [127]	Specific and efficient binding to hepcidin	Double-blind, placebo-controlled, randomized phase I dose-escalation clinical trial	HD patients (*n* = 24)	Intravenous administration of a single ascending dose of PRS-080#22 (2–8 mg/kg) or placebo. 28 days	Increased serum iron levels and transferrin saturation. Reduced serum hepcidin levels	Treatment was safe and well tolerated
LY3113593 monoclonal antibody [129]	Prevention of BMP 6 binding to its receptor, suppressing hepcidin expression	Placebo-controlled, randomized clinical trial	HD patients (*n* = 8) receiving usual doses of ESA and iron	Single intravenous administration of LY3113593 (150 mg) or placebo (*n* = 6 and *n* = 2, respectively). 84 days	Increased Hb and serum iron levels. Decreased serum ferritin and hepcidin levels	Good safety and tolerability
LY2928057 monoclonal antibody [129]	Blocking the interaction of hepcidin with ferroportin, allowing a continued iron efflux from exporting tissues into plasma	Placebo-controlled, randomized clinical trial	HD patients (*n* = 28) discontinuing ESA and/or iron therapies at randomization time	Intravenous administration to HD patients of placebo (*n* = 7) or LY2928057 (310 mg, *n* = 6; 600 mg, *n* = 11; 1000 mg, *n* = 4), every 2 weeks for 3 doses. 6 weeks	Increased serum iron, transferrin saturation and hepcidin levels; decreased serum ferritin. Decrease over time in Hb concentration	Good safety and tolerability
Erythroferrone [131]	Inhibition of BMP signalling required for transcription of hepcidin	Pre-clinical study in vivo	Mouse model of adenine-induced CKD	Transgenic augmentation of erythroferrone compared to wild-type littermates. 8 weeks	Increased Hb by approximately 2 g/dL and iron mobilization. Modestly improved renal function	Overt adverse effects not observed
Transferrin receptor 2-targeted therapy [136]	Deletion of Tfr2 in the liver hampers hepcidin production, while in the hematopoietic compartment it increases erythroid EPO sensitivity	Pre-clinical study in vivo	Mouse model of adenine-induced CKD	Simultaneous inactivation of hepatic and erythroid Tfr2 compared to wild-type littermates. 8 weeks	Amelioration of anemia with enhanced red blood cell production and iron supply without increasing EPO levels	No adverse clinical renal events
KY1070 antibody [137]	Inhibition of BMP6-induced hepcidin expression	Pre-clinical study in vivo	Mouse model of adenine-induced CKD	Intraperitoneal administration of IgG4 isotype control (*n* = 5), KY1070 (3 mg/kg; *n* = 6), Darbepoetin alfa (10 mg/kg; *n* = 6), or both (*n* = 6) 4 weeks	Monotherapy with KY1070 or darbepoetin ameliorated Hb levels. Combination therapy normalized Hb levels and RBC count, inducing the strongest increase in plasma iron levels and a significant increase in transferrin saturation	Not reported

CKD, chronic kidney disease; HD, hemodialysis; BMP, bone morphogenetic protein; ESA, erythropoiesis stimulating agent; Hb, hemoglobin; Tfr2, Transferrin receptor 2; EPO, erythropoietin; RBC, red blood cell.

#### 3.3.4. Anti-Inflammatory Interleukin-Targeting Monoclonal Antibodies

Promising results for the treatment of anemia in CKD patients with chronic inflammation have been obtained so far with interleukin-targeting monoclonal antibodies.

*Ziltivekimab*, a potent neutralizing, fully human monoclonal antibody against the IL-6 ligand, was first evaluated in a randomized phase 1 clinical trial in NDD CKD patients with evidence of systemic inflammation. One-time treatment with ziltivekimab subcutaneously was safe and highly effective at suppressing hs-CRP [143]. A randomized phase 1/2 trial examined the effect of ziltivekimab in HD patients with inflammation and hypo-responsiveness to ESA therapy [144]. All participants had rs855791 single nucleotide polymorphism in the TMPRSS6 gene (this is present in approximately 80% of the population), hypothesized to increase susceptibility to the inflammatory effects of IL-6. Ziltivekimab therapy led to an improvement in anemia markers, including Hb concentration, a decline in ESA requirement, and an improvement in inflammatory biomarkers [144]. Using data from the randomized RESCUE trial [145], a recent exploratory analysis assessed the effect of ziltivekimab on Hb and iron homeostasis in patients with CKD stage 3–5 not on dialysis and systemic inflammation [146]. The study terminated early due to the coronavirus disease 2019 pandemic, and the data presented reached up to 12 weeks. Ziltivekimab safely induced a significant increase in Hb levels and in iron metabolism biomarkers [146].

These pilot clinical trials suggest ziltivekimab may represent a safe and effective approach for managing anemia in CKD [147]. A recent exploratory synthesis of early-phase trials [143,144,146] (*n* = 337) showed that IL-6 inhibition by ziltivekimab over 12 weeks was associated with high certainty with a significant increase in Hb (0.87 g/dL) as compared to placebo, and a generally favorable safety profile, generating hypotheses relevant to future research [148].

In addition, given the pivotal role of IL-6 in atherothrombosis [149], IL-6 inhibition by ziltivekimab might benefit individuals at high atherosclerotic risk, such as CKD patients. The RESCUE trial [145] and the RESCUE 2 trial [150] assessed the effects of ziltivekimab on multiple biomarkers of inflammation and thrombosis in participants with stage 3–5 CKD (*n* = 264 and *n* = 36, respectively). In both studies, ziltivekimab decreased serum levels of hs-CRP, serum amyloid A and fibrinogen, with subsequent resolution towards the baseline value after two weeks of drug washout. These phase 2 clinical studies set the stage to design the ongoing ZEUS (Ziltivekimab Cardiovascular Outcomes Study) trial (NCT05021835), a large-scale trial addressing whether targeting IL-6 can enable reductions in cardiovascular events in CKD patients (eGFR 20–60 mL/min/1.73 m^2^) with known atherosclerosis and inflammation [151].

Another high-affinity, humanized monoclonal antibody targeting the IL-6 ligand is *Clazakizumab*, which is currently undergoing clinical development for antibody-mediated kidney transplant rejection [152]. The POSIBIL6ESKD is a double-blind, randomized, placebo-controlled trial combining dose-finding (phase 2b, completed) and cardiovascular outcome (phase 3, ongoing) in HD patients with cardiovascular disease and/or diabetes and systemic inflammation (NCT05485961). In phase 2b, clazakizumab treatment significantly reduced serum hs-CRP levels compared to placebo by 90% or more, meeting the primary outcome. The safety profile of clazakizumab appeared acceptable and consistent with its mechanism of action [153]. A prespecified secondary analysis of the trial in the 127 HD patients showed that although no change in Hb levels was found at 12 weeks (possibly related to a change in ESA dose that was not restricted by protocol), the use of clazakizumab reduced the need for ESA initiation or dose escalation, improved serum iron metabolism biomarkers, and reduced serum hepcidin [154]. Phase 3 of the POSIBIL6ESKD trial is evaluating the effect of clazakizumab 5 mg on the primary outcome of either cardiovascular death or nonfatal myocardial infarction.

*Canakinumab* is a human monoclonal antibody that targets IL-1β, examined in the CANTOS (Canakinumab Anti-Inflammatory Thrombosis Outcomes Study) trial for the prevention of atherosclerotic events [155]. In 1875 patients with CKD stage 3 at study entry, over a median follow-up period of 3.7 years, allocation to canakinumab significantly reduced the risk of major cardiovascular events, with no adverse clinical renal events [156]. Despite efficacy, at present the drug lacks regulatory approval for this indication. A post hoc analysis of CANTOS explored whether IL-1β inhibition with canakinumab might slow the onset of incident anemia and improve Hb levels among patients with prevalent anemia at trial entry [157]. The administration of canakinumab reduced the incidence of anemia, including in CKD stage 3 patients. Among patients with baseline anemia, canakinumab increased Hb levels, but CKD as the cause of anemia was not specified. Moreover, treatment with canakinumab was associated with a slightly albeit significantly increased risk for nonopportunistic fatal infections. Further interventional trials are therefore necessary to evaluate canakinumab therapy in anemia of CKD.

Though they are not yet available for clinical use, anti-inflammatory therapies using monoclonal antibodies can be beneficial for use against anemia of CKD, reducing its prevalence and increasing Hb levels (Table 4). By improving anemia and lowering inflammation, IL-6-targeting agents are promising options to reduce the elevated morbidity and mortality of CKD. Long-term data on their efficacy and safety are needed prior to large-scale clinical use.

## 4. Emerging Approaches to Anemia Across Kidney Replacement Therapy Modalities

Kidney replacement therapy represents a heterogeneous clinical setting for anemia management, as the relative contributions of EPO deficiency, inflammation, iron-restricted erythropoiesis, blood loss, and treatment-related factors may differ between hemodialysis, peritoneal dialysis, and kidney transplantation.

Most of the evidence regarding investigational strategies reviewed herein has been generated in patients receiving HD. This population is particularly relevant because persistent inflammation, iron-restricted erythropoiesis, dialysis-related blood losses, and ESA hyporesponsiveness frequently coexist. Nutritional interventions and early clinical studies of activin traps have predominantly been evaluated in HD patients, although the available evidence remains heterogeneous and generally insufficient to support their routine use. Among the investigational approaches, studies with IL-6-targeting monoclonal antibodies (ziltivekimab and clazakizumab) have shown favorable effects on inflammatory and iron-related biomarkers and, in some studies, reduced ESA requirements. HD currently represents the kidney replacement modality in which the potential role of novel anemia treatments is best characterized, although long-term efficacy and safety data remain necessary for most investigational strategies.

In contrast, patients receiving peritoneal dialysis are substantially underrepresented in studies evaluating emerging therapies for CKD anemia. This represents an important evidence gap, since PD differs from HD with respect to residual kidney function, blood losses, inflammatory burden, iron administration strategies, and ESA requirements. Specific evidence in PD for most of the adjunctive and investigational approaches discussed in this review is currently very limited or absent. Consequently, the efficacy observed in HD patients should not automatically be extrapolated to PD patients, and dedicated studies are warranted.

Post-transplant anemia represents a distinct and multifactorial condition in kidney transplantation. In addition to impaired graft function and iron deficiency, contributing factors may include inflammation, infections, immunosuppressive and other medications, nutritional deficiencies, and reduced EPO production [158]. Evidence regarding the emerging approaches considered in this review is particularly scarce in kidney transplant recipients. There is currently insufficient evidence to define a role in post-transplant anemia for the novel strategies discussed herein, such as EPO mimetic peptides, activin traps, hepcidin-targeted therapies and cytokine-targeting monoclonal antibodies.

Thus, future clinical trials should include modality-specific analyses and dedicated peritoneal dialysis and transplant populations to determine whether the efficacy and safety of novel approaches differ across kidney replacement therapies.

## 5. Conclusions

The search for the most effective and at the same time safest therapy for anemia in patients with CKD has always been a focus for nephrologists, especially because dialysis and renal transplants have allowed a notable prolongation of life for these patients, such that the quality of life has become an even more important objective for them than simple survival.

The basic therapy so far has been the correction of iron deficiency. More recently, thanks to new iron preparations, there has been a shift from reactive treatments to proactive treatments of iron deficiency, and the use of iron has also reduced the need for ESAs and HIF-PHIs and their doses. This approach has also been supported by cardiology studies that have demonstrated a favorable effect of iron therapy in non-anemic heart failure patients. We know well how frequent this pathology is in CKD patients, particularly those on dialysis, given the progressive aging of the population starting dialysis treatment. Undoubtedly, ESA therapy has revolutionized the lives of these patients, who have gone from surviving to living, albeit with significant limitations. In addition to the favorable effects, limitations to the use of therapy with ESAs have also emerged, particularly regarding cardiovascular, thrombotic, and neoplastic risks, in addition to their hypo-responsiveness in the presence of an inflammatory state.

For this reason, iron administration has been used not only to correct its deficiency and improve frequent heart failure, but also to reduce the doses of ESAs and therefore mitigate the possible side effects.

With the aim of finding alternative therapies to ESA therapy, natural remedies and drugs that primarily reduce inflammation, a known important factor that reduces the effectiveness of ESAs, have been sought. Overall, none of these approaches have been found to have a very impactful effect, but they can all be considered adjuvants in the correction of anemia (Table 5). However, adjuvant pharmacological (pentoxifylline, L-carnitine, androgens) and nutritional interventions cannot currently be recommended for routine treatment, except when correcting a documented deficiency or another specific underlying cause.

Interestingly, SGLT2i were found to be able to increase hemoglobin levels. The reason for this interest lies in the fact that these drugs work alongside RASIs in cardiovascular protection, and a risk of using RASIs, in addition to the increase in potassium levels, is the worsening of anemia. Both these important side effects are brilliantly overcome by SGLT2i, even if caution should be exercised in their use in patients with preserved renal function and polycystic disease due to the potential risk of polycythemia with associated thrombotic risks, which are also increased by possible dehydration due to their use. It is presently unknown whether SGLT2i also maintain their antianemic effects in patients with advanced CKD.

In the last 15 years, several therapeutic attempts have been made to suppress hepcidin, with limited success. This is probably because hepcidin regulation is redundant, governed by competing signals—iron, inflammation, erythropoiesis, and hypoxia—and limited by significant safety concerns, rendering single-pathway interventions often incomplete or transient.

New avenues are being explored for the treatment of anemia, including that of CKD, and inflammation is the main target, considering its frequent and progressive presence in CKD with the progressive deterioration of renal function, and its relevant role in hypo-responsiveness to treatment with ESAs. We are also awaiting, with much interest, the results regarding their general effect on the most relevant complications of CKD, including atherosclerosis and cardiovascular damage.

## Figures and Tables

**Table 2:** Clinical studies with EPO mimetic peptides and activin traps in CKD anemia.

Agent (Ref. )	Mechanism of Action	Study Design	Participants (Number)	Intervention, Study Duration	Main Results	Safety
EPO Mimetic Peptides						
Pegmolesatide [113]	Binding to and activation of EPO receptor	Multicenter, single-arm, open-label, dose-finding phase II trials	NDD-CKD patients (*n* = 62 included in the FAS and 51 in the PPS) and HD patients (*n* = 62 in FAS and 51 in PPS)	Pegmolesatide sc once every 4 weeks. Initial doses 0.025 mg/kg, 0.05 mg/kg, or 0.08 mg/kg adjusted to achieve a target Hb range of 10.0–12.0 g/dL. 24 weeks	Increased Hb levels above baseline at four weeks. All groups achieved the target Hb range at the 18th week of the trial (by the fifth administration)	Treatment was well tolerated and adverse effects proved mild and manageable
Pegmolesatide [114]	Binding to and activation of EPO receptor	Randomized, open-label, active-comparator, non-inferiority phase III trial	Patients on maintenance dialysis (HD or PD) for at least 12 weeks, treated with pegmolesatide (*n* = 233 in the PPS) or epoetin alfa (*n* = 114 in the PPS)	Pegmolesatide sc once every four weeks or epoetin alfa 1–3 times per week, to maintain Hb levels between 10 and 12 g/dL. 52 weeks	Mean change in Hb levels from baseline to the efficacy evaluation period (weeks 17–24) comparable in the two groups, proving non-inferiority of pegmolesatide to epoetin alfa. These results were maintained during the 52-week study period	Treatment was well tolerated. Safety profile similar between the two groups; hypertension the most common adverse effect
Pegmolesatide [115]	Binding to and activation of EPO receptor	Rrandomized, open-label, active-controlled, noninferiority phase 3 study	Stage 3–5 CKD patients without previous dialysis or ESA treatment within 12 weeks before randomization (115 in the pegmolesatide group and 58 in the epoetin alfa group)	Pegmolesatide 0.04 mg/kg sc once every four weeks or epoetin alfa 6000 IU 1–2 times per week, to maintain Hb levels between 10 and 12 g/dL. Doses of study drugs could be adjusted to achieve the target Hb range. 52 weeks	Mean change in Hb levels from baseline to the efficacy evaluation period (weeks 17–24) comparable in the two groups, proving non-inferiority of pegmolesatide to epoetin alfa. These results were maintained during the 52-week study period	Safety outcomes similar between groups. No cardiovascular concern
Activin Traps						
Sotatercept [116]	Binding to activin and other members of the TGF-beta superfamily that adversely affect hematopoiesis	REN-001: Single-blind, placebo-controlled, randomized study to evaluate anemia correction after ESA suspension	HD anemic patients with ESA hypo-responsiveness (*n* = 43)	Sotatercept (0.3 to 0.7 mg/kg) or placebo sc every 4 weeks. 200 days	Achievement of target Hb response (>10 g/dL) more frequently achieved in patients treated with sotatercept (dose-dependently) than placebo group. Reduced need for rescue therapies such as ESA or transfusion as the sotatercept dose increased	Acceptable safety and tolerability profiles
Sotatercept [116]	Binding to circulating activin and other members of the TGF-beta superfamily that adversely affect hematopoiesis	REN-002 study—open-label, randomized trial evaluating Hb maintenance after switching from a prior ESA	HD anemic patients with ESA hypo-responsiveness (*n* = 50)	Sotatercept (intravenous, 0.1 to 0.4 mg/kg; subcutaneous 0.13 to 0.50 mg/kg) every 2 weeks. 99 days	Sixteen (32%) patients maintained predefined target Hb (10–12 g/dL) without rescue medication. Similar proportions of patients used rescue therapy in the intravenous and sc dose groups	Acceptable safety and tolerability profiles

EPO, erythropoietin; CKD, chronic kidney disease; NDD, non-dialysis dependent; FAS, full analysis set; PPS, protocol compliant set; HD, hemodialysis; sc, subcutaneous; Hb, hemoglobin; PD, peritoneal dialysis; ESA, erythropoiesis stimulating agent; TGF-beta, transforming growth factor-beta.

**Table 4:** Clinical studies with anti-inflammatory interleukin-targeting monoclonal antibodies in CKD patients.

Agent, Study	Study Design	Participants (Number)	Intervention, Study Duration	Main Results	Safety
Ziltivekimab Nowak et al., 2021 [143]	Randomized phase I dose-escalation trial	NDD-CKD patients with eGFR 20–60 mL/min/1.73 m^2^ and hs-CRP > 2 mg/L (*n* = 12)	Ziltivekimab 5, 15, or 50 mg subcutaneous once 12 weeks	↓ hs-CRP	No serious adverse events reported
Ziltivekimab Pergola et al., 2021 [144]	Placebo-controlled, randomized phase I/II trial	HD patients with ↑ IL-6 (*n* = 61)	Placebo or Ziltivekimab (2, 6, or 20 mg) intravenous every 2 weeks 12 weeks	Improvement of anemia markers (Hb, serum iron, total iron binding capacity, transferrin saturation). Decreased ESA requirements. Improvement of inflammation biomarkers (hs-CRP, serum amyloid A, fibrinogen)	No dose-limiting toxicity. Some concern for risk of infection
Ziltivekimab Pergola et al., 2024 [146]	Placebo-controlled, randomized phase II trial	Stage 3–5 CKD patients (GFR > 10 and <60 mL/min/1.73 m^2^) with systemic inflammation (hs-CRP ≥ 2 mg/L)	Placebo or Ziltivekimab (7.5, 15, or 20 mg) subcutaneous once every 4 weeks (*n* = 46 for each study group) 12 weeks	Significant increases in Hb levels and serum iron metabolism biomarkers (iron levels, total iron binding capacity, transferrin saturation). No change in serum ferritin and hepcidin levels.	Safety outcomes similar between groups
Clazakizumab Chertow et al., 2024 [153]	Placebo-controlled, randomized-phase IIb dose-finding trial	HD patients with cardiovascular disease and/or diabetes and systemic inflammation (hs-CRP ≥ 2 mg/L	Placebo (*n* = 31) or Clazakizumab (2.5, 5 or 10 mg) intravenous every 4 weeks (*n* = 32 per each dose group) 12 weeks	Significant reduction in serum levels of hs-CRP and inflammatory biomarkers (fibrinogen, amyloid A, secretory phospholipase A2, and lipoprotein(a)); significant increase in serum albumin	Safety profile acceptable and consistent with Clazakizumab’s mechanism of action
Clazakizumab Neuen et al., 2025 [154]	Prespecified secondary analysis of Chertow’s study [153]	HD patients with cardiovascular disease and/or diabetes and systemic inflammation (hs-CRP ≥ 2 mg/L)	Placebo (*n* = 31) or Clazakizumab (2.5, 5 or 10 mg) intravenous every 4 weeks (*n* = 32 per each dose group) 12 weeks	No change in Hb levels. Reduced need for ESA initiation or uptitration. Improved serum iron metabolism biomarkers (transferrin saturation, iron, total iron-binding capacity). Reduced serum hepcidin.	Safety profile acceptable and consistent with Clazakizumab’s mechanism of action
Canakinumab Ridker et al., 2018 [156]	Double-blind, placebo-controlled, randomized trial (CANTOS trial)	Stage 3 CKD patients (*n* = 1875) with history of myocardial infarction and hs-CRP ≥ 2 mg/L	Placebo or Canakinumab (50, 150 or 300 mg) subcutaneous every 3 months. Mean follow-up period of 3.7 years	Reduced risk of major adverse cardiovascular events by 18%, and by 32% in patients achieving on-treatment hs-CRP levels < 2 mg/L	No adverse clinical renal events
Canakinumab Vallurupalli et al., 2020 [157]	Post hoc exploratory analysis of CANTOS	8683 CANTOS participants without anemia at trial entry and 1303 with prevalent anemia at trial entry, including CKD patients (stage 3 at trial entry)	Placebo or Canakinumab (50, 150 or 300 mg) subcutaneous every 3 months. Mean follow-up period of 3.7 years	Significant risk reduction in incident anemia, including in participants with eGFR < 60 mL/min/1.73 m^2^ (hazard ratio, 0. 80)	Increased risk for fatal infections. Mild thrombocytopenia and neutropenia

CKD, chronic kidney disease; NDD, non-dialysis dependent; eGFR, estimated glomerular filtration rate; hs-CRP, high sensitivity C-reactive protein; HD, hemodialysis; IL-6, Interleukin-6; Hb, hemoglobin; ESA, erythropoiesis stimulating agent; CANTOS (Canakinumab Anti-Inflammatory Thrombosis Outcomes Study) trial.

**Table 5:** Overview of the level/maturity of available evidence on emerging and adjunctive strategies for CKD anemia.

Therapeutic Option	CKD 3–4	CKD 5 NDD	CKD 5D	Current Evidence/Perspective
SGLT2 inhibitors	+++	++	-	Most immediately applicable strategy: consistent erythropoietic effect, though antianaemic effect in advanced CKD still uncertain
Pentoxifylline	+	+	++	Possible adjunct in patients with inflammatory phenotype and ESA hypo responsiveness; evidence remains limited
L-carnitine	-	-	++	Possible ESA-sparing adjunct in HD patients with ESA hypo responsiveness; evidence not definitive
Zinc supplementation	+	+	++	Potentially useful only in zinc-deficient patients, particularly HD patients receiving ESA; requires monitoring of serum zinc and copper levels
Lactoferrin	-	+	++	Preliminary clinical evidence in iron restricted/inflammatory anemia; further RCTs required
Gut microbiota modulators	+	+	++	Promising nutritional adjunct in patients with inflammatory or dysbiotic phenotype; quality evidence heterogeneous and moderate
Natural source polysaccharides	?	?	?	Predominantly preclinical/very low-level clinical evidence
Pegmolasetide	++	+++	+++	Promising advanced-development strategy evidenced by phase III trials in NDD and dialysis patients requiring ESA therapy; need for longer-term and broader-population data
Activin traps	-	-	++	Biological/clinical signal in dialysis patients with ESA hyporesponsiveness; however, studies are limited, and no further CKD development is currently planned
Hepcidin-targeted therapies	+	+	++	Strong mechanistic rationale but mixed clinical results; most programs discontinued or at early stage
Interleukin-6-targeting therapies	++	++	+++	Among the most promising investigational approaches, particularly in CKD with systemic inflammation and/or ESA hyporesponsiveness; long-term safety/efficacy and outcome data needed
Interleukin-1 beta inhibition	+	?	?	Suggestive evidence in inflammatory phenotype but CKD-anemia-specific evidence insufficient

CKD, chronic kidney disease; NDD, non-dialysis dependent; 5D, dialysis-dependent; SGLT2, sodium-glucose co-transporter 2; ESA, erythropoiesis stimulating agent; HD, hemodialysis; RCT, randomized controlled trial. Symbols for the level of evidence in different CKD stages: +++, strongest promise/evidence for the indicated CKD setting; ++, potentially useful/promising; +, preliminary or selected-patient potential; -, little/no relevant evidence or applicability; ?, insufficient evidence.

## Data Availability

No new data were created or analyzed in this study. Data sharing is not applicable to this article.

## Abbreviations

The following abbreviations are used in this manuscript:

CKD	Chronic Kidney Disease
DF	Dietary Fiber
eGFR	estimated Glomerular Filtration Rate
EMP	EPO Mimetic Peptide
EPO	Erythropoietin
ESA	Erythropoiesis-stimulating Agent
Hb	Hemoglobin
Hct	Hematocrit
HIF-PHIs	Hypoxia-inducible Factor Prolyl Hydroxylase Inhibitors
hs-CRP	High sensitivity C-reactive Protein
NDD	Non-dialysis Dependent
SGLT2i	Sodium Glucose co-transporter 2 Inhibitor
SIRT1	Sirtuin1
Tfr2	Transferrin Receptor 2
TGF-β	Transforming Growth Factor-β
TNF-α	Tumor Necrosis Factor alpha
